# Supplementary material for: Chemical and Genotypic Variations in Aniba rosiodora from the Brazilian Amazon Forest
Source: Molecules. 2020 Dec 25;26(1):69. doi: 10.3390/molecules26010069 (PMC7794742; doi:10.3390/molecules26010069)
Supplement: Supplementary file 1 [file molecules-26-00069-s001.pdf]

Supplementary Material

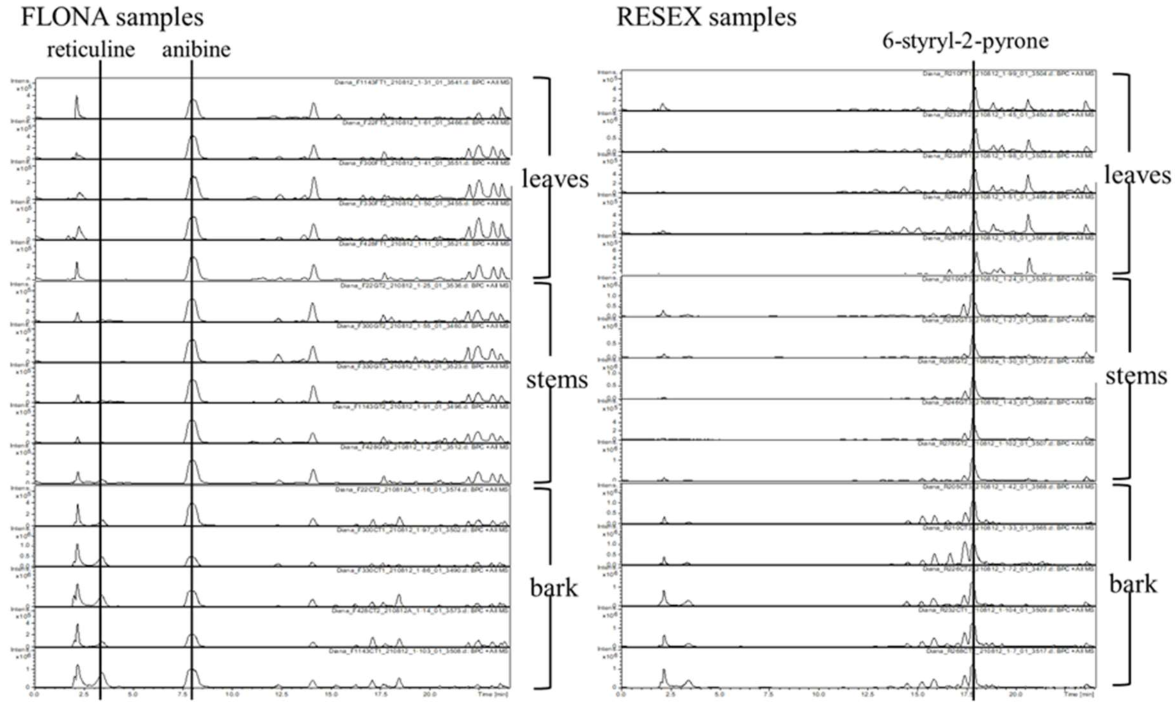

Figure S1. Mass spectra of crude methanol extract of different parts of *Aniba* tree from FLONA and RESEX. The peaks of anibine, reticuline and 6-styryl-2-pyrone are highlighted.

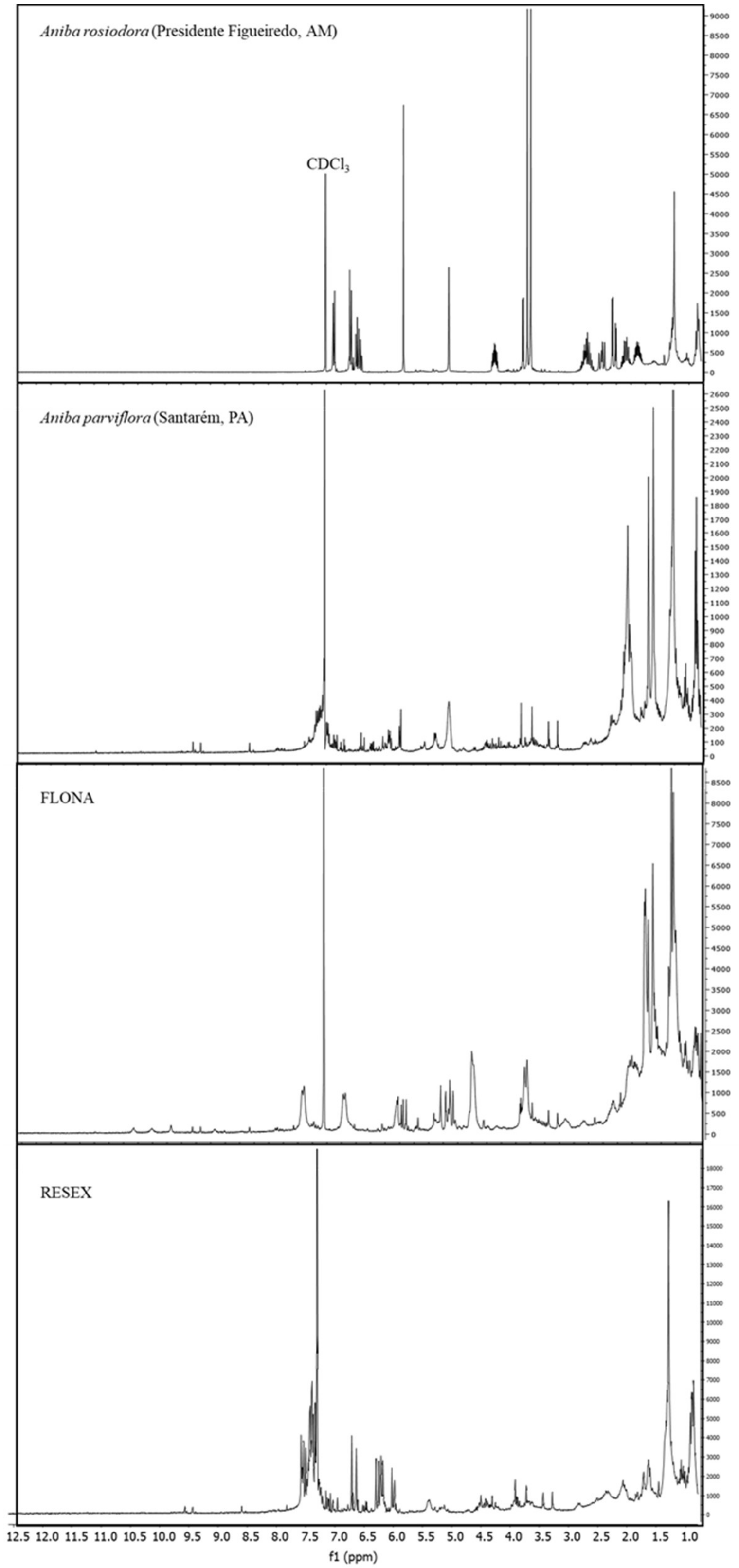

Figure S2: 1H-NMR spectra data from crude methanolic extract of FLONA, RESEX, *A. parviflora* and *A. rosiodora* leaves.

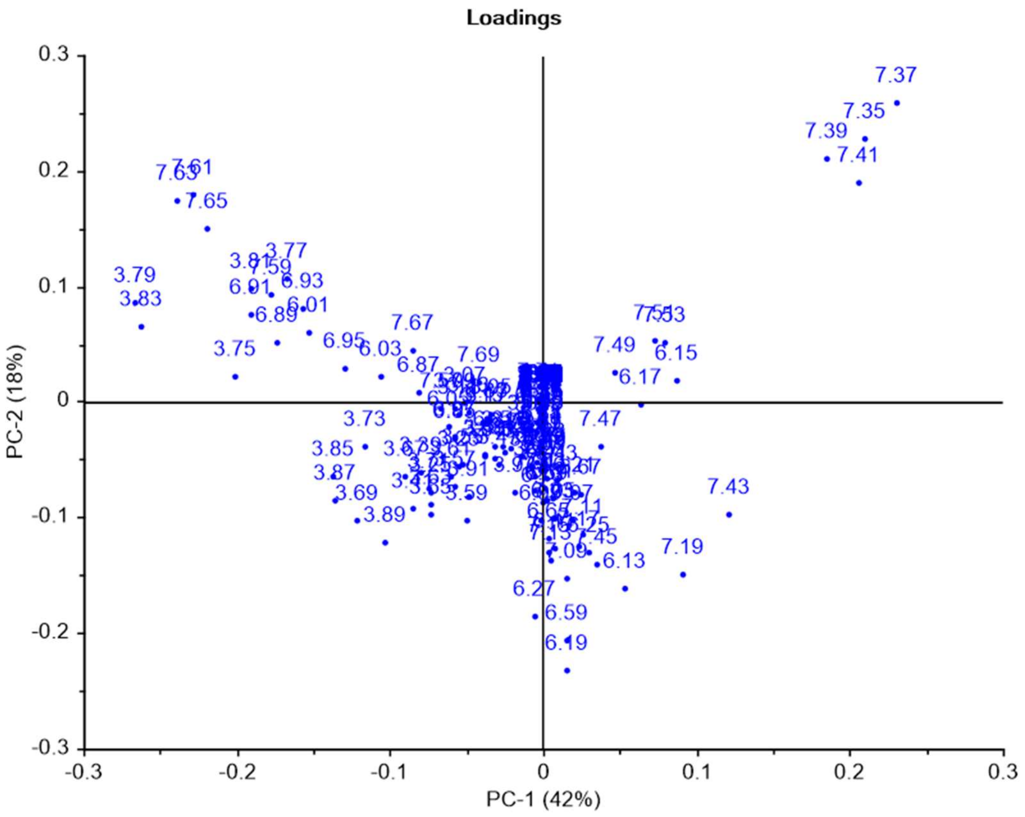

Figure S3: Loading plot from the PCA analysis using ^{1}H NMR spectra data from crude methanolic extract of FLONA, RESEX, *A. parviflora* and *A. rosiodora* leaves.
